# Radiographic and safety details of vertebral body stenting: results from a multicenter chart review

**DOI:** 10.1186/1471-2474-14-233

**Published:** 2013-08-08

**Authors:** Peter Diel, Christoph Röder, Gosia Perler, Thomas Vordemvenne, Matti Scholz, Frank Kandziora, Sebastian Fürderer, Soren Eiskjaer, Gianluca Maestretti, Robert Rotter, Lorin Michael Benneker, Paul Friedhelm Heini

**Affiliations:** 1 Department of Orthopaedic Surgery, Inselspital, University Hospital Bern, Freiburgstrasse, 3010 Bern, Switzerland; 2 MEM Research Center, Institute for Evaluative Research in Orthopaedic Surgery, University of Bern, Stauffacherstrasse 78, CH-3014 Bern, Switzerland; 3 Department of Trauma Surgery, University Hospital Münster, Domagkstraße 5, 48149 Münster, Germany; 4 Center for Spinal Surgery and Neurotraumatology, BGU Hospital Frankfurt, Friedberger Landstraße 430, 60389 Frankfurt, Germany; 5 Department of Spine Surgery, Mutterhaus der Borromäerinnen Hospital Trier, Feldstraße 16, 54290 Trier, Germany; 6 Department of Spine Surgery, Aalborg University Hospital, Hobrovej18–22, 9100, Aalborg, Denmark; 7 Department of Spine Surgery, Cantonal Hospital Fribourg, Chemin des Pensionnats 2, 1708 Fribourg, Switzerland; 8 Department of Spine Surgery, University Hospital Rostock, Schillingallee 35, 18057 Rostock, Germany; 9 Spine Center, Sonnenhof Hospital, Buchserstrasse 30, 3006 Bern, Switzerland

**Keywords:** Vertebral fracture, Vertebral augmentation, Vertebral body stenting, Stentoplasty, Osteoporosis

## Abstract

**Background:**

Up to one third of BKP treated cases shows no appreciable height restoration due to loss of both restored height and kyphotic realignment after balloon deflation. This shortcoming has called for an improved method that maintains the height and realignment reached by the fully inflated balloon until stabilization of the vertebral body by PMMA-based cementation. Restoration of the physiological vertebral body height for pain relief and for preventing further fractures of adjacent and distant vertebral bodies must be the main aim for such a method. A new vertebral body stenting system (VBS) stabilizes the vertebral body after balloon deflation until cementation. The radiographic and safety results of the first 100 cases where VBS was applied are presented.

**Methods:**

During the planning phase of an ongoing international multicenter RCT, radiographic, procedural and followup details were retrospectively transcribed from charts and xrays for developing and testing the case report forms. Radiographs were centrally assessed at the institution of the first/senior author.

**Results:**

100 patients (62 with osteoporosis) with a total of 103 fractured vertebral bodies were treated with the VBS system. 49 were females with a mean age of 73.2 years; males were 66.7 years old. The mean preoperative anterior-middle-posterior heights were 20.3-17.6-28.0 mm, respectively. The mean local kyphotic angle was 13.1°. The mean preoperative Beck Index (anterior edge height/posterior edge height) was 0.73, the mean alternative Beck Index (middle height/posterior edge height) was 0.63. The mean postoperative heights were restored to 24.5-24.6-30.4 mm, respectively. The mean local kyphotic angle was reduced to 8.9°. The mean postoperative Beck Index was 0.81, the mean alternative one was 0.82. The overall extrusion rate was 29.1%, the symptomatic one was 1%. In the osteoporosis subgroup there were 23.8% extrusions. Within the three months followup interval there were 9% of adjacent and 4% of remote new fractures, all in the osteoporotic group.

**Conclusions:**

VBS showed its strengths especially in realignment of crush and biconcave fractures. Given that fracture mobility is present, the realignment potential is sound and increases with the severity of preoperative vertebral body deformation.

## Background

In case of failed conservative therapy with analgesia, bed rest or adapted physiotherapy, percutaneous vertebroplasty (VP) and balloon kyphoplasty (BKP) are well approved and established methods for treating painful vertebral body fractures especially in osteoporotic but also in trauma and tumor patients [1,2]. Whilst VP is mainly applied for fracture stabilization without the intention of direct deformity correction, BKP was developed for better height restoration and realignment as well as for decreased extrusion risks based on creating an intravertebral cavity with walls of impacted cancellous bone. The superiority of BKP over VP in height restoration and realignment probably cannot be disputed [3] but loss of vertebral body height and realignment after balloon deflation have called for a further improved method maintaining conditions reached with the fully inflated balloon until cement has been delivered.

Restoration of the physiological vertebral body height for pain relief and for preventing further fractures of adjacent und distant vertebral bodies must be the main aim for such a method [4-6].

Therefore the Vertebral Body Stenting System (VBS) was developed which uses a balloon-catheter-mounted stent that is expanded by inflating a balloon inside the vertebral body. With its intrinsic mechanical stability, the expanded rigid stent construct keeps the created cavity open after balloon deflation until PMMA-based cement is injected and has cured [7].

The aim of the current analysis was to describe clinical and radiographic results of the new endovertebral stenting system obtained by chart review of the first 100 cases.

## Methods

### Technical data

The VBS system (Synthes GmbH, Oberdorf, Switzerland) consists of a balloon-expandable metal stent mounted on a balloon-catheter, of which two are inserted bilaterally into the vertebral body and simultaneously inflated with contrast-saline solution, under pressures up to 30 atm, to symmetrically expand both stents. The stent implants consist of a cobalt–chromium alloy commercially known as L605, also extensively used in the cardiovascular area, e.g. in coronary and peripheral artery stenting. The expanded stent comes along pre-crimped on the balloon (Ø 4.2 mm in its unexpanded state), and is gradually expanded to its final diameter. At the time of this study a 20 mm and 15 mm long version was available, which has meanwhile been complemented with a 13 mm version for the upper thoracic levels cranial to Th10. The laser-cut mesh pattern keeps spreading apart until fracture reduction is radiographically satisfying and/or the maximum stent diameter of 17 mm is reached. After the balloon-assisted stent expansion is sufficient and/or complete, the balloons are deflated and retrieved, leaving both expanded stents behind to keep the restored height. Finally, PMMA cement is injected into the mesh structures to produce a stent-reinforced cement implant within the treated vertebral body [7].

Information was retrospectively transcribed from charts and radiographs with clinical and radiographic case report draft forms at the University hospital Bern, Switzerland; the Sonnenhof hospital Bern, Switzerland; the University hospital Muenster, Germany; BG Unfall-klinik Frankfurt, Germany; hospital Mutterhaus der Borromäerinnen, Trier, Germany; Aalborg Fysiurgiskeclinic, Denmark; the University hospital of Rostock, Germany, and the cantonal hospital Fribourg, Switzerland. The data were then entered into the MEMdoc online database of the Institute for Evaluative Research in Orthopaedic Surgery at the University of Bern [8]. Radiographs were centrally assessed at the institution of the first/senior author. No patient outcomes like VAS, ODI or EQ-5D were recorded. Due to the anonymized retrospective and observational nature of the study, no IRB approval was obtained.

The study protocol for the ongoing international multicenter RCT was partially applied for data collection of the current analysis:

– Inclusion criteria were fractures of one to three contiguous vertebral bodies, AO classification fracture types A1.x or A.3.1, locations between Th10 –L5, a loss of height above 15%, a positive MRI with fracture edema and clinical findings including local back pain > 4 (VAS 0–10) correlating with the location of the fracture.

– Exclusion criteria were asymptomatic fractures or stable fractures that responded well to conservative treatment; diffuse pain without MRI evidence of an active fracture as shown by the fracture edema; systemic or local infections and severe bleeding disorders.

– The following parameters were assessed:

○ patient demographics

○ fracture characteristics (type, age, morphology, radiology)

○ procedural details

○ perioperative and followup complications

○ revision and reintervention surgeries

○ realignment

**Figure 1:**
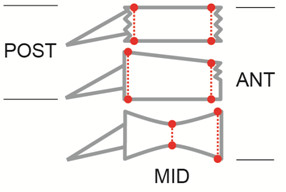
**Beck Index (BI) and Alternative Beck Index in different fracture types.** BI Crush fractures: Ant Height/Post Height. BI Wedge fractures: Ant Height/Post Height. Alternative BI biconcave fractures: Ant Height/Mid Height

Fracture type was described with the AO classification [9] and a subjective morphologic term (wedge, crush, biconcave). Beck Index (BI) and absolute heights (mm) with comparison to reference heights was favored over simply reporting kyphotic angles because crush fractures can display a simple overall height loss without kyphotic deformation and biconcave fractures can have a severe central height loss with relative maintenance of anterior and posterior edge heights without a kyphotic deformation either. For the latter case an alternative Beck Index was introduced for demonstrating improvements in mid vertebral body heights; the traditional Beck Index cannot show any pre- to postoperative changes in vertebral body morphology of those types of fractures (Figure 1).

### Statistical analysis

Chi-square and Wilcoxon rank-sum tests were used for unpaired comparisons of binomial and continuous variables, respectively. The Wilcoxon signed rank test was used for within-patient pre- to postoperative comparison of continuous variables. Spearman’s correlation coefficient was used for calculating the correlation between preoperative Beck Index and the pre- to postoperative Beck Index change. The level of significance was set to 0.05. Statistical analyses were performed using the software package SAS 9.2 (SAS Institute Inc, Cary, NC).

### Sensitivity analysis

Despite striving for standardized postoperative imaging in a routine clinical setting we did still receive slightly different height and angulation measurements in case of several follow-ups for one and the same case. In those cases we calculated the minimum and maximum possible height restoration and realignment scenarios and a final average of these values.

### Non-mover/poor-mover analysis

We defined those cases as non-movers that did not show a postoperative realignment of more than 0.05 BI units despite an obvious potential for fracture mobility based on preoperative imaging. We defined those cases as poor-movers that did not show a postoperative realignment of more than 0.1 BI units despite an obvious potential for fracture mobility based on preoperative imaging.

## Results

100 patients (62 with osteoporosis, marked as OP) with a total of 103 (63 OP) fractured vertebral bodies were treated with the VBS system. 49 (37 OP) patients were females with a mean age of 73.2 (76.3 OP) years (range 41.1-87.1, OP 55.6-87.1 years), 51(OP 25) were males with a mean age of 66.7 (OP 71) years (range 35.4-91, OP 51.6-91 years). The time between incidence of fracture occurrence and treatment was less than one week in 24 (OP 16.3) %, up to three weeks in 66 (OP 58) % and more than three weeks in 34 (OP 42) %. 62% were osteoporotic fractures, 34% traumatic and 2% lytic ones. 25 (OP 33.9) % of fractures were localized between Th10-12, 57 (OP 46.8) % between L1-3, and 18 (OP 19.4) % at L4 or L5. There were 41 (OP 38.3) % of AO fractures type A3.1, 30 (OP 33.3) % of A1.2, 18 (OP 18.3) % A1.3 and 11 (OP 10) % A1.1. Applying a different morphologic classification there were 53.6 (OP 50) % crush, 26.8 (OP 25) % wedge, and 19.8 (OP 25) % biconcave fractures.

The procedural details of the intervention have been well described by Klezl and Muto [10,11]. In the current series, a transpedicular approach was chosen in 94 (OP 97) %, a lateral extrapedicular one in 6 (OP 3) % of the cases. The mean balloon inflation pressure was 23 (OP 22.6) Atm (range 12–30, OP 13–30 Atm) and the mean filling volume was 10 ml of cement (range 5-17 ml, same for OP). In two vertebral bodies a mono-pedicular approach was inadvertently chosen against the recommendations by the manufacturer. In 55 (OP 60) % of cases Synthes’ Vertecem “NF” PMMA-based cement and in 45 (OP 40) % other PMMA-based cements were applied. At the time of the first 100 VBS cases where Vertecem “NF” PMMA-based cement was being used, its viscosity was monitored by a viscometer (“Viscosafe”, Anton Paar GmbH, Austria) indicating when to start safe manual cement injection with pre-filled cement syringes. Not in all cases the viscometer was used by the surgeons.

### Incidence of intraoperative complications

1. Intraoperative complications with stent or balloon catheter placement:

There was 1 balloon and 2 stent misplacements, but without any negative clinical consequences.

2. Intraoperative complications during stent expansion / balloon inflation:

There were 6 recorded stent maldeployments that were all attributable to an immobile fracture or sclerosis zone. No other complications were seen, provided the max. balloon volume and pressure limitations were observed. If not, there is a risk of balloon leakage that does, however, not necessarily carry clinical complications.

3. Intraoperative complications during cement injection:

There were 36 (OP 18) cement extrusions in 30 (OP 15) vertebral bodies. 16 (OP 6) extrusions went into the paraspinal soft tissues, 11 (OP 7) into the disc, 4 (OP 3) into paravertebral vessels, 3 (OP 1) epidurally and 2 (OP 1) into the foramen. 1 neurocompression was treated with an intraoperative decompression. The extrusion rate based on the number of treated levels was hence 29.1 (OP 23.8) %, the symptomatic extrusion rate was 1%.

### Follow-up/revision

There were 128 follow-up and 12 revision/re-intervention forms completeable (3 revisions, 8 new interventions, 1 combined revision and new intervention), with a mean time of 119 days for the follow-ups (range 3–468 days) and 48 days for the revisions (range 10–141 days). 16 patients did not have a recorded follow-up or revision. Table 1 details the indications for the reintervention or revision procedures.

**Table 1:** Reintervention/revision procedures and indications

**Type of intervention**	**Indication**	**No of patients**
Revision	Neurocompression with recurrence of pain	3
New intervention	New adjacent fracture(s)	4
New intervention	New remote facture(s)	4
Revision & new intervention	Neurocompression and new adjacent fracture	1

### Complications

There was a recurrence of symptoms at the same level in 12 cases and a sintering of the treated vertebral body in 8 cases. 2 new radiculopathies were treated with a decompression and rigid stabilization. There were 5 new cranial and 5 new caudal adjacent vertebral fractures and 3 new cranial and 1 new caudal remote vertebral fracture which corresponds with 9% of adjacent and 4% of remote fractures (2 patients had adjacent and remote fractures combined). All new fractures occurred in the osteoporotic patient group.

### Analysis of cases with an adjacent or remote fracture during follow-up

Patient demographics, pre- as well as postoperative local kyphotic angles and occurrence of intradiscal extrusions were compared in patients with and without new vertebral fractures. Table 2 depicts significant and non-significant differences between the patient groups.

**Table 2:** Patient and fracture characteristics of cases with and without new fractures

	**New fracture**	**No new fracture**	**p-value**
Age (mean)	76 (69–88) yrs	69 (35–91) yrs	0.322
Gender (% female)	81.8%	48%	0.036*
LKA preop (mean)	16° (46°-1°)	12.6° (50°-0°)	0.687
LKA postop (mean)	10° (15°-1°)	8.7°(27°- 0°)	0.471
Intradiscal extrusions	0%	11.8%	n.a

*significant difference.

### Height restoration and realignment

#### Preoperative overall values

**Figure 2:**
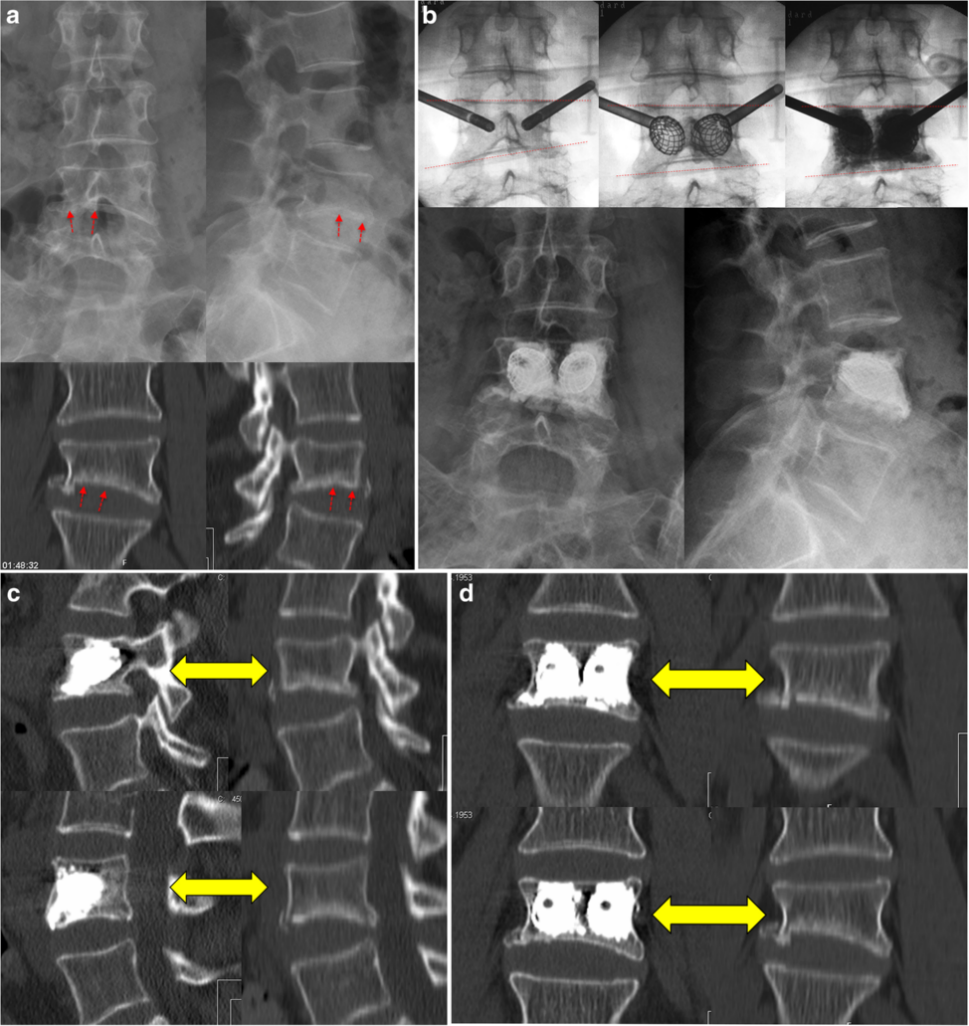
**Case presentation.** This 63 years old female presents after a minor car accident. Clinically the patient presents with right sided leg pain as soon as she gets up, however motor function is ok. The imaging studies depict an atypical fracture of L4 (Figure 2a), with an impression of the lower endplate. Obviously there is a foraminal stenosis at L4-L5 which provokes the leg pain. Because of the neurologic symptoms a surgical treatment was advocated. Instead of an open intervention a stentoplasty procedure was performed. The correction achieved is well visible by the intra- and postoperative pictures (dashed lines, Figure 2b). Compared to preoperatively the postoperative CT scan confirms an important correction that could be achieved by the stent and the well performed cement augmentation that stabilizes the vertebral body (Figures 2c,d).

The mean anterior-middle-posterior heights were 20.3-17.6-28.0 (OP 19.4-16.4-26.4) mm, respectively. The mean local kyphotic angle, i.e. the angle between both endplates was 13.1 (OP 10.1)°. The mean anterior-middle-posterior heights of the next healthy cranial or caudal reference levels were 33.0-31.0-33.0 (OP 31.8-29.2-31.8) mm, respectively; their mean local kyphotic angle was 0.2 (OP 0.1°). The mean preoperative Beck Index (anterior edge height divided by posterior edge height) was 0.73 (OP 0.74), the mean alternative Beck Index (middle height divided by posterior edge height) was 0.63 (OP 0.62) (Figure 1). Figure 2 depicts an exemplary case history.

#### Overall values at last radiographic follow-up (~6 months postop)

The mean anterior-middle-posterior heights were 24.5-24.6-30.4 (OP 23.9-23.4-29.1) mm (p < 0.0001, p < 0.0001, p = 0.0027) respectively. The mean local kyphotic angle, i.e. the angle between both endplates was 8.9° (OP 7.5°) (p < 0.0001, OP: p < 0.0001). The mean postoperative Beck Index was 0.81 (OP 0.83)(p < 0.0001), the mean alternative Beck Index was 0.82 (OP 0.81) (p < 0.0001).

Taking the respective healthy referent level as 100%, the ant-mid-post heights were improved from an average 61.7%-59.3%-87% (OP 57.1%-55.6%-82.9%) preoperative to an average 78.9%-85.6%-97.3% (OP 80.6%-86.7%-99.0%) of original height postoperative. The correlation between preoperative Beck Index and pre- to postoperative Beck Index improvement was −0.692 (OP −0.728)(p < 0.0001) and −0.732 (OP same)(p < 0.0001) for the alternative one. This means that the smaller the preoperative Beck Index, i.e. the larger the kyphotic deformity, the larger the pre-to postoperative BI difference, i.e. the extent of realignment that is achieved with VBS.

#### Stratification by fracture morphology

**Figure 3:**
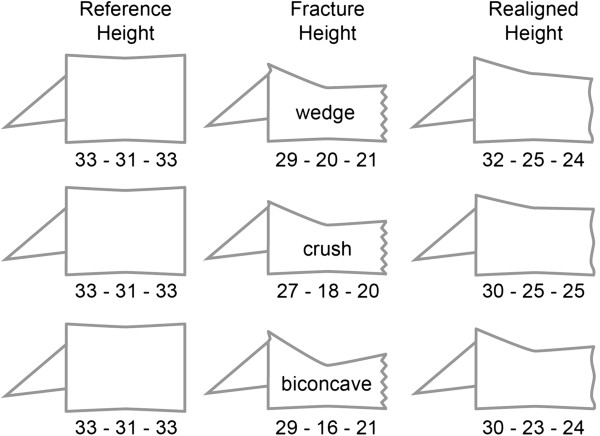
Authentic relations of reference heights and mean group heights (mm) for the three fracture types (as classified by the treating surgeon). Images scaled but downsized.

**Figure 4:**
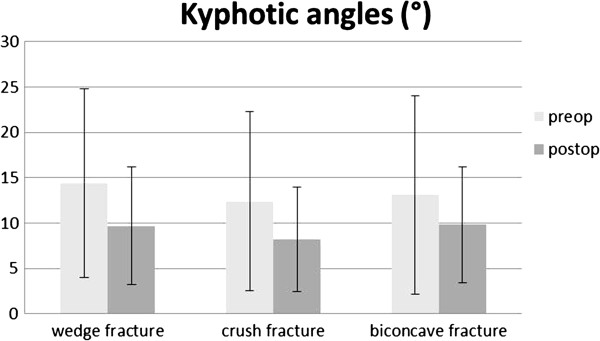
Mean (SD) kyphotic angles (°) before and after surgery for the three fracture types.

Tables 3, 4, 5 and Figures 3, 4 show the size and shape characteristics of the fractured and realigned vertebral bodies stratified by the three subjective morphologic deformation types.

**Table 3:** Pre- and postoperative height of vertebral bodies with wedge fractures

**Height (mm)**	**Mean preop**	**% of referent height**	**Mean postop**	**% of referent height**	**p-value**
	**All/OP**	**All/OP**	**All/OP**	**All/OP**	**All/OP**
anterior	20.7/20.0	63.0/60.7	23.8/24.5	73.9/79.6	0.019/0.039
middle	19.7/18.5	64.2/62.3	25.2/23.3	84.9/84.5	<0.0001/0.023
posterior	29/27.5	88.3/85.0	31.7/30.7	98.6/100.7	0.008/ 0.312*

*not significant.

**Table 4:** Pre- and postoperative height of vertebral bodies with crush fractures

**Height (mm)**	**Mean preop**	**% of referent height**	**Mean postop**	**% of referent height**	**p-value**
	**All/OP**	**All/OP**	**All/OP**	**All/OP**	**All/OP**
anterior	20.1/18.4	60.8/54.1	25.1/23.6	81.5/82.2	<0.0001/<0.0001
middle	17.5/16.1	58.4/53.6	25.1/ 23.7	87.6/90.7	<0.0001/<0.0001
posterior	27.3/25.0	85.4/79.5	29.9/27.9	97.2/99.8	0.019/0.012

**Table 5:** Pre- and postoperative height of vertebral bodies with biconcave fractures

**Height (mm)**	**Mean preop**	**% of referent height**	**Mean postop**	**% of referent height**	**p-value**
	**All/OP**	**All/OP**	**All/OP**	**All/OP**	**All/OP**
anterior	20.6/21.3	62.6/60.7	23.6/24.1	78.3/77.7	0.023/0.127*
middle	15.6/15.3	54.6/52.5	22.6/22.6	80.0/79.4	0.002/0.016
posterior	28.6/28.7	89.9/89.5	30.2/30.1	95.5/94.9	0.549*/0.643*

*not significant.

### Wedge fractures

For wedge fractures (N = 23, OP N = 13 with complete radiographic data), the preoperative Beck Index was 0.73 (OP 0.75), the alternative Beck Index was 0.68 (OP same). At the last follow-up it was 0.76 (OP 0.80) and 0.79 (OP 0.76) (p = 0.324, p = 0.0062, respectively. For OP p = 0.7422, p = 0.2031). The preoperative local kyphotic angle was 14.4 (OP 14.7)°, postoperative it was 9.7 (OP 8.7)° (p = 0.007; OP p = 0.1250).

### Crush fractures

For crush fractures (N = 46, OP = 26 with complete radiographic data), the Beck Index was 0.74 (OP same), the alternative Beck Index was 0.64 (OP same). At the last follow-up it was 0.85 (OP same) and 0.85 (OP 0.86)(p < 0.0001 for both indices and OP). The preoperative local kyphotic angle was 12.4 (OP 12.5)°, postoperative it was 8.2 (OP 7.4)° (p = 0.0003; OP p = 0.0034).

### Biconcave fractures

For biconcave fractures (N = 16, OP N = 11 with complete radiographic data), the Beck Index was 0.72 (OP 0.73), the alternative Beck Index was 0.53 (OP 0.51). At the last follow-up it was 0.79 (OP 0.8) and 0.75 (OP same) (p = 0.0012, p = 0.0001, respectively. OP p = 0.0195, p = 0.001).The preoperative local kyphotic angle was 13.1 (OP 12.3)°, postoperative it was 9.8 (OP 8.7)° (p = 0.0034; OP p = 0.0117).

### Sensitivity analysis

**Figure 5:**
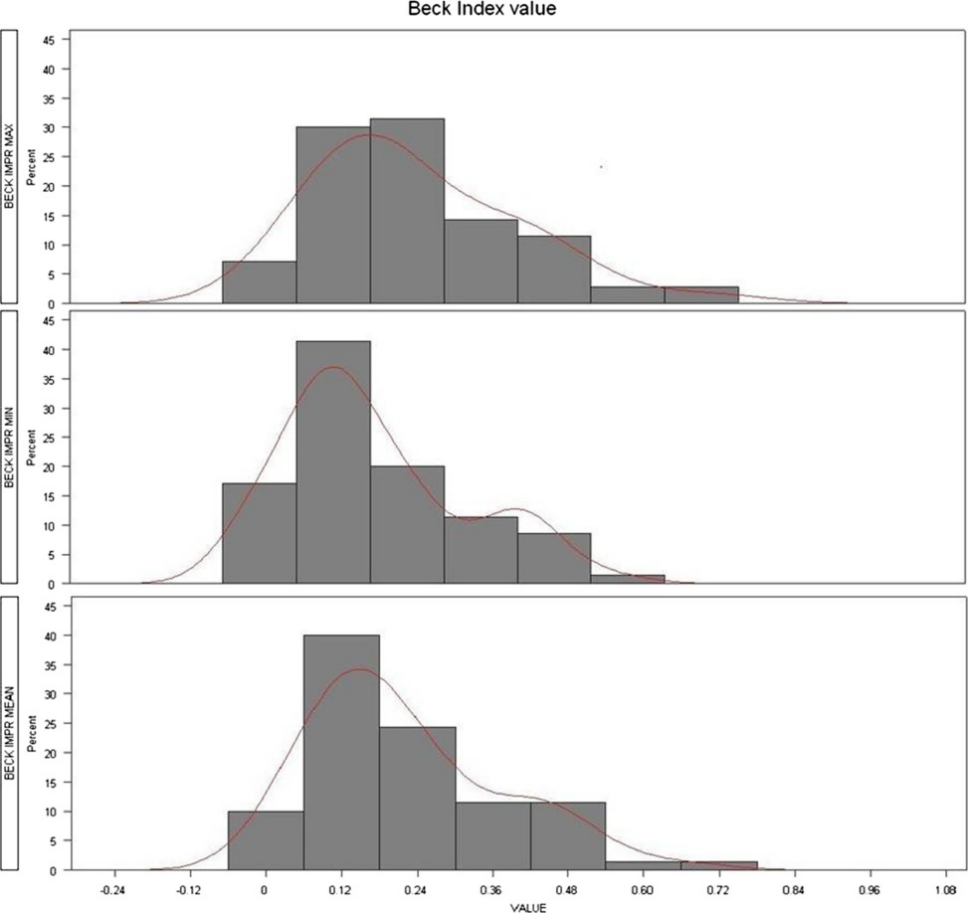
Maximum-minimum-average scenarios of pre- to postoperative Beck-Index improvement.

The above cited overall mean postoperative Beck Index of 0.81 (OP same) was improved to 0.84 (OP 0.85) in the maximum and to 0.77 (OP 0.78) in the minimum scenario. The resulting pre- to postoperative improvements were hence 0.23 (OP 0.24) BI units in the maximum, 0.16 (OP same) units in the minimum, and 0.2 (OP same) units in an average scenario. The curves of the maximum and minimum case scenarios reveal that about 35-50% of cases have a potential for a BI improvement of ~0.3-0.5 units (Figure 5).

### Non-movers, poor movers

Non-mover or poor mover instances can occur if the fracture is consolidated or the bone too hard for any balloon inflation or stent expansion at all, despite positive fracture edema on MRI. There were four non-movers in our sample and excluding them from the analysis the average improvement in Beck Index rose from 0.2 to 0.25 units. There were six poor-movers in our sample in addition to the four non-movers, and excluding those ten cases from the analysis the average improvement in Beck Index rose from 0.2 to 0.26 units. Hence, in about 10% of cases we had a not appreciable height or alignment restoration. Looking at all cases of type “movers” i.e. full balloon inflation / complete stent expansion possible, with a wedge shaped fracture morphology, the local kyphotic angle was improved by a mean of 10° (17.7° preop to 7.6° postop). In the non- and poor-movers group, this improvement was only 4° (15.8° preop to 11.8° postop).

## Discussion

The current case series presents the short-term results of a very early series of the first 100 cases treated with the VBS system. In the meantime four other studies have presented clinical and radiographic outcomes of the VBS system, but all patient samples were considerably smaller (~ 50–20 cases) [10-13]. Overall, good restoration of vertebral body height and alignment were obvious, but a direct translation of realignment into a significantly reduced rate of adjacent vertebral fractures, as suggested in the biomechanical literature, could not be shown in our series [14], and much larger case numbers will be needed for a conclusive assessment of this aspect.

VBS was developed for overcoming the weakness of the BKP system in incompletely maintaining the restored vertebral body realignment and height that is achieved with a fully inflated ballon [15,16]. This weakness of BKP leaves a considerable one third of treated cases without an appreciable height restoration [2]. The percentage and the overall extent of realignment shall be improved with VBS based on the principles of balloon kyphoplasty and vascular stenting. Using VBS, the stent remains within the newly created vertebral cavity so the balloon can be removed after deflation while preventing the vertebral body from collapsing, so that, in an ideal scenario, a virtually physiological vertebral body height and shape can be restored and preserved. In their recent RCT, Werner et al. did not find significant differences in vertebral kyphosis correction between BKP and VBS. Both systems achieved around 4.6° of mean reduction [13]. Despite a sufficiently powered analysis and presumably well balanced fracture characteristics, preoperative kyphotic deformity angles of the two groups were not reported, which, according to our findings, have an influence on the reduction potential of a fracture. Moreover, stratified results by fracture type were not reported, which may have revealed differences between the two therapies. Thaler et al. could only achieve an average 3.5° reduction of vertebral kyphosis in 27 patients, but the mean preoperative Beck Index of 0.87 implies a smaller reduction potential than that of our group (preop 0.73), which may explain the better reduction of 4.2° in the current study [12].

Using a different unit for describing re-alignment but kyphotic angles, Maestretti et al. reported Beck Index improvements of 0.14 in their traumatic BKP case series treated with calciumphosphate cement [17] and Krüger et al. of about 0.07 in their series of osteoporotic incomplete burst fractures [18]. However, in the latter series the preoperative BI values were around 0.8, whilst those in the Maestretti series were 0.7 and hence better comparable with the extent of VB deformation in our series. The overall BI improvement we found was 0.2, and even 0.26 in an idealized scenario ignoring the non-moving and poor-moving fractures. These values do also make clear that they can only be achieved in fractures with preoperative BI values of about 0.7 or lower.

The indication for applying a vertebral augmentation method from the meanwhile available spectrum of “simple” vertebroplasty with no intrinsic mechanical method for height restoration but patient positioning, to balloon kyphoplasty and vertebral body stenting should not only be based on fracture type, patient characteristics and extrusion risks but also on the extent of vertebral body deformation. An only mildly deformed fracture has a generally small realignment potential, and fractures with Beck Indices around 0.8 and local kyphotic angles of 8.5° [18] are probably more suitable for BKP or even VP than fractures with Beck Indices < = 0.7 and local kyphotic angles of 17° [17] where VBS can develop its full realignment potential. As shown in Figure 5, about a third and up to half of the fractures had mean BI improvements of 0.3-0.5, but such extent of restoration can only be achieved with a corresponding preoperative deformity. If not given, the realignment potential of therapies such as VBS and other implant based augmentation technologies is limited by a “ceiling effect”, i.e. restoration of BI to values greater 1.0 is largely impossible and the resulting overcorrection clinically not meaningful. In addition, we could show that reports of Beck Indices and local kyphotic angles are influenced by fracture morphology and should hence be described separately. In our case series there were about 20% biconcave fractures and the relation between vertebral body middle height and posterior wall height, herein introduced as alternative Beck Index is the most appropriate way of describing these fractures. The local kyphotic angle or the original Beck index reflecting the relation between anterior and posterior vertebral height are less suited for a description of biconcave fractures. The anterior-posterior wall relations deal with the important local and the resulting segmental kyphotic deformity which is, based on biomechanical considerations, responsible for increased risks of new adjacent and distant vertebral fractures and patients’ postural decompensation in the kyphotic plane [14].

Klezl pointed out that kyphosis correction with VBS was better in the traumatic group where even reduction of the fractured endplate with the stent could be achieved with possible implications on future performance of injured discs in young patients [10]. Research suggests that a vertebral trauma and especially a fractured endplate can cause disc cell apoptosis and disc degeneration [19,20]. The anterior spinal column, especially the fragmented superior endplate could be well reconstructed by the stent provided that it was inserted accurately. Our results confirm these observations where the alternative BI was improved from 0.53 to 0.75 in biconcave but also from 0.64 to 0.85 in crush fractures while it only increased from 0.68-0.79 in wedge shaped fractures. Endplate and mid vertebral height restoration are new aspects in minimally invasive fracture treatment whereas improved realignment and decreased cement leakage was the original goal of the balloon kyphoplasty principle.

Looking at all moving wedge shaped fractures, the local kyphotic angle was even improved by 10°, from preoperative 17.7° to 7.6°. Diel et al. report average improvements of 4° with VP [21] and Hulmes reports an average of 6.6° improvement for BKP and VP in his systematic review [2]. In contrast, Papanastassiou et al. reported 4.8° kyphotic angle change from baseline for BKP and only 1.7° for VP in their systematic review of randomized and non-randomized controlled studies. Such comparisons highlight the potential of VBS. However, a translation into significantly reduced adjacent fracture rates could not be deduced from our data yet. A 9% rate of new fractures is lower than the 10.4% after BKP but higher than the 8.4% after VP as reported by Papanastassiou [3]. Considering our analysis of cases with new fractures after surgery, the sex distribution in these groups should always be reported since female gender seems to represent a risk factor.

The overall 29% cement extrusion rate we observed is comparable with BKP rates if assessed in an independent fashion. The FREE study reported a 27% extrusion rate whereas the systematic analysis of Hulme et al. calculated an only 9% rate based on published literature [2,22]. In many reports, however, authors have assessed their own extrusions which probably lead to a gross underreporting. Thaler et al. reported a 25.5% extrusion rate in their VBS series and Werner et al. had a 20% minor and 10% major leakage rate. In both studies, a viscometer was used [12,13]. The final rate of symptomatic extrusions in our series was 1% which is lower than the 2.6% in VP and comparable with the 1.3% in BKP reported by Hulme [2]. Klezl had 2 asymptomatic leakages in 20 treated vertebral bodies and Muto had none [10,11]. Klezl pointed out the more difficult situation in osteoporotic bone where a slight and careful overfilling of cement should be aimed at for achieving a good interdigitation of cement with bone [10]. Considering the 50% osteoporotic fractures in our series and its 23.8% extrusion rate, the cementation challenge in this group seems to have been well met, this despite most surgeons in our series probably not having passed their VBS learning curve yet. New cementing techniques like radiofrequency kyphoplasty may help to further reduce leakage rates. Kurth et al. reported a respectably low rate of only 15.5% in a multicenter study with 186 treated vertebral bodies [23]. High viscosity cement is another promising option for leakage prevention, which has mostly been applied and assessed in vertebroplasty. Georgy found only 8% of moderate or severe leakage in a chart/xray review of 66 treated levels [24], but this figure is not easy to compare with other reports that have not graded leakage into none, mild, and more severe types. In the meantime, a higher viscosity cement is also available for VBS (Vertecem “V+”) and future studies will have to show if it can help reduce leakage rates to levels comparable with the above cited percentages.

## Conclusions

The current case series demonstrated the promising performance of VBS in fracture reduction and realignment if indications are correctly made. One important aspect regarding indication making is the extent of vertebral body deformation and morphology, the other is true fracture mobility in seemingly mobile fractures on pre-operative imaging. Another way of assessing any potential for deformity correction is to use a trial balloon as a fracture mobility and expansion simulation tool prior to stenting, to confirm the intra-operative fracture mobility and restoration potential before any stent is inserted.

## Competing interests

The following authors have competing interests: C Röder, S Fürderer and P Heini are consultants of Synthes. The manuscript processing charge will be covered by Synthes.

## Authors’ contributions

PD is a resident who was responsible for project administration (radiographic measurements, data entry) and manuscript drafting. CR is a senior researcher who planned and conceptualized the project and planned and supervised the data analysis. He assisted in manuscript writing. GP is a statistician who performed all statistical and data analyses. TV is a surgeon who contributed cases and data and reviewed and corrected the drafted manuscript. MS is a surgeon who contributed cases and data and reviewed and corrected the drafted manuscript^.^ FK is a surgeon who contributed cases and data and reviewed and significantly helped improve the drafted manuscript^.^ SF is a surgeon and the inventor of VBS. He contributed cases and data and reviewed and corrected the drafted manuscript SE is a surgeon who contributed cases and data and reviewed and corrected the drafted manuscript. GM is a surgeon who contributed cases and data and reviewed and corrected the drafted manuscript. RR is a surgeon who had previously conducted and published a biomechanical analysis of VBS. He also contributed cases and data and reviewed and corrected the drafted manuscript. LB is a surgeon who contributed cases and data and reviewed and corrected the drafted manuscript. PFH is a surgeon and senior consultant. He supervised the project design, execution, and manuscript drafting. He also contributed the largest number of cases. All authors read and approved the final manuscript.

## Pre-publication history

The pre-publication history for this paper can be accessed here:

http://www.biomedcentral.com/1471-2474/14/233/prepub
